# Transduction of the MPG-tagged fusion protein into mammalian cells and oocytes depends on amiloride-sensitive endocytic pathway

**DOI:** 10.1186/1472-6750-9-73

**Published:** 2009-08-26

**Authors:** So-Jung Kwon, Kyuyong Han, Suhyun Jung, Jong-Eun Lee, Seongsoon Park, Yong-Pil Cheon, Hyunjung Jade Lim

**Affiliations:** 1 Department of Biomedical Science & Technology IBST Konkuk University 1 Hwayang-dong, Kwangjin-gu, Seoul 143-701, Korea; 2 Department of Chemistry, Center for NanoBio Applied Technology, Institute of Basic Sciences, Sungshin Women's University, Seoul 136-742, Korea; 3 School of Biological Sciences and Chemistry, Sungshin Women's University 136-742 Korea

## Abstract

**Background:**

MPG is a cell-permeable peptide with proven efficiency to deliver macromolecular cargoes into cells. In this work, we examined the efficacy of MPG as an N-terminal tag in a fusion protein to deliver a protein cargo and its mechanism of transduction.

**Results:**

We examined transduction of MPG-EGFP fusion protein by live imaging, flow cytometry, along with combination of cell biological and pharmacological methods. We show that MPG-EGFP fusion proteins efficiently enter various mammalian cells within a few minutes and are co-localized with FM4-64, a general marker of endosomes. The transduction of MPG-EGFP occurs rapidly and is inhibited at a low temperature. The entry of MPG-EGFP is inhibited by amiloride, but cytochalasin D and methyl-β-cyclodextrin did not inhibit the entry, suggesting that macropinocytosis is not involved in the transduction. Overexpression of a mutant form of dynamin partially reduced the transduction of MPG-EGFP. The partial blockade of MPG-EGFP transduction by a dynamin mutant is abolished by the treatment of amiloride. MPG-EGFP transduction is also observed in the mammalian oocytes.

**Conclusion:**

The results show that the transduction of MPG fusion protein utilizes endocytic pathway(s) which is amiloride-sensitive and partially dynamin-dependent. Collectively, the MPG fusion protein could be further developed as a novel tool of "protein therapeutics", with potentials to be used in various cell systems including mammalian oocytes.

## Background

Cell-permeable or cell-penetrating peptides (CPPs) are considered a promising method to deliver macromolecular cargoes into live cells across lipid bilayers. These peptides commonly bear stretches of basic amino acids. Many CPPs have been shown to deliver oligonucleotides or siRNA efficiently across cell membrane with or without covalent conjugation [1]. As for the delivery of proteins, expression and purification of CPP-fusion protein in a single step produces more stable form of cargo for in vitro and in vivo uses. The Protein Transduction Domain (PTD) from TAT protein of the human immunodeficiency virus (HIV), is known to deliver large proteins up to ~120 kDa into cells in the form of fusion protein [2,3]. Thus, it has been in the forefront of protein delivery and is being widely used to deliver various proteins for functional experiments [4]. Other CPPs have been tested for their efficiency for protein delivery by cross-linking, by simple mixing, or as a fusion protein form [5-7].

While the list of applicable CPPs is expanding, recent works have been focused on identifying the mechanisms of cellular uptake of CPPs. These studies generally utilize arrays of endocytosis inhibitors to identify specific endocytic pathway involved in the uptake [8-11]. These works provided evidence that the uptake of TAT PTD, polyarginine, and other peptides is dependent on the lipid rafts-mediated macropinocytosis, "the cell-drinking" process.

MPG is a designed CPP comprised of two independent domains [12]. The first 17 amino acids of the N-terminus is derived from glycine-rich region of the viral gp41 [13] and the hydrophilic C-terminus from nuclear localization signal (NLS) of the SV40 large T antigen [14]. The original MPG peptide is acetylated at the N-terminus and synthesized with a cysteamide group at the C-terminus. This form was used to deliver siRNA or oligonucleotide and the effectiveness was proven in several cell systems [12,15]. A recent report showed that the initial interaction of MPG peptide with the cell surface uses negatively charged glycosaminoglycans. Furthermore, the mechanism of MPG peptide-mediated delivery of nucleic acids seems to involve Rac1-dependent remodeling of actin network within the cell [16]. However, the potential for MPG as a carrier of protein cargoes has not been investigated.

The present investigation was initiated to identify CPPs which can be effectively used for the protein delivery in the form of CPP-fusion proteins. We chose 8 known CPP sequences and prepared CPP-EGFP fusion proteins [5,6,12,17-20]. Our initial screening unveiled the efficient transduction of modified MPG-EGFP fusion proteins into various cell lines and thus we focused on identifying the mechanism of cellular uptake of MPG-EGFP. We report herein that the uptake of MPG-fusion proteins utilizes specific endocytic pathway which is sensitive to amiloride and partially dependent on dynamins.

## Results

### High transduction efficiency of MPG-EGFP fusion protein in mammalian cells

**Figure 1:**
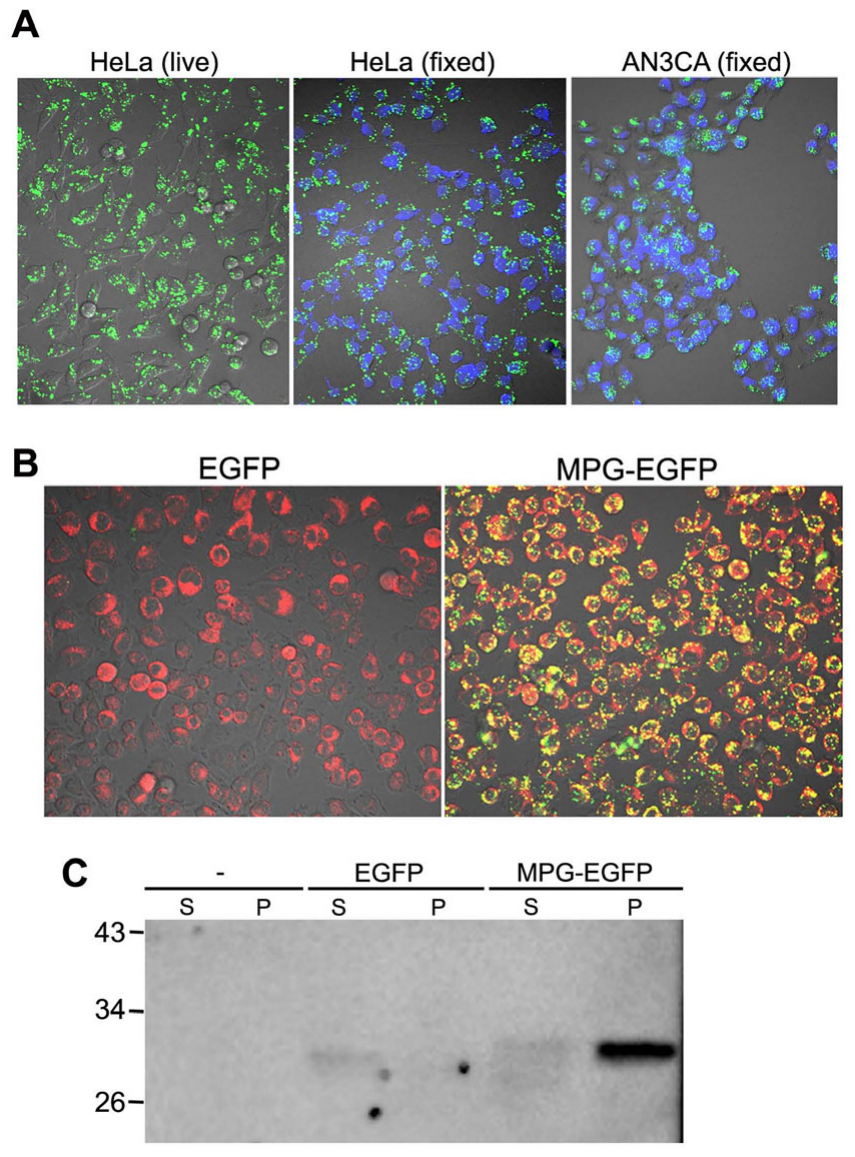
**Efficient transduction of the MPG-EGFP fusion protein into various cell lines**. A. 40 μg/ml MPG-EGFP was added to AN3CA or HeLa cells. NIH3T3, BV2, 293T, and HT29 were also tested (data not shown). MPG-EGFP exhibits a punctate vesicular pattern in the cytoplasm in cells. Fixation of the cells with 4% PFA (fixed) did not alter the subcellular distribution of MPG-EGFP. Cells were all observed under a confocal microscope. Fixed cells were counterstained with TO-PRO-3 iodide (shown in blue, 1:500). B. MPG-EGFP is mostly present in the endosomes. Cells were stained with 5 μg/ml FM4-64, a general marker of endocytosis (shown in red), and visualized under a confocal microscope without fixation. Overlapping of MPG-EGFP signal and FM4-64 staining generates a yellow fluorescence. C. Vesicle fractionation was performed using HeLa cells treated with EGFP or MPG-EGFP for 1 hr. -, no treatment; EGFP, 40 μg/ml EGFP; MPG-EGFP, 40 μg/ml MPG-EGFP; S, supernatant containing the cytosolic fraction; P, pellet containing the intracellular vesicles. Western blotting was performed with anti-GFP antibody.

We initially chose 8 CPPs that have not been used to deliver protein cargoes in the form of fusion proteins (Table 1), and prepared CPP-EGFP fusion proteins. These CPPs, in peptide forms, had all been shown effective in delivering oligonucleotides or nucleic acids into cells when used as a mixture. As N-terminal tags of EGFP recombinant proteins, however, most of these CPPs did not seem to enter cells efficiently (data not shown). Among the tested CPPs, MPG-EGFP entered the cells and exhibited a punctate vesicular pattern of EGFP fluorescence (Figure 1A). Efficient transduction of MPG-EGFP was confirmed in various cell lines, including AN3CA, 293T, NIH3T3, F9, BV2, and HT29. Treatment of MPG-EGFP at 40, 80, or 120 μg/ml for more than 24 hr did not cause any significant cytotoxicity (all above 93% survival rate).

Representative figures are shown in Figure 1A. Intracellular localization of MGP-EGFP was mostly cytoplasmic vesicular patterns reminiscent of endosomal vesicles. Since the fixation of cells reportedly affects the subcellular localization of CPPs [21], we compared the subcellular localization of MPG-EGFP in live and fixed cells. As shown in Figure 1A, no significant redistribution of MPG-EGFP signal in the cells was noted. To examine if MPG-EGFP proteins are present within endosomal vesicles, we stained the MPG-EGFP-treated HeLa cells with a general fluorescence marker of endocytosis FM4-64 [10]. As shown in Figure 1B, most of the MPG-EGFP overlaps with the endosomal staining of FM4-64 (red). Localization of MPG-EGFP in intracellular vesicles was further confirmed by the vesicle fractionation procedure. By using the differential centrifugation, the vesicle fractionation procedure separates the particulate fractions containing most of the subcellular vesicles from the cytosolic supernatant. As shown in Figure 1C, the vesicle fraction of the MPG-EGFP treated cells showed a clear immunoreactive EGFP, confirming the vesicular localization of MPG-EGFP. The immunoreactive EGFP was detected in the cytosolic fraction at a low intensity. The result shows that MPG-EGFP proteins are mainly localized in endosomes. The confocal live imaging clearly demonstrated that MPG-EGFP signals are present within multiple endosomes. These vesicles dynamically move around and a small number of them fuses or disappears during several minutes of observation (see Additional file 1).

**Table 1:** CPPs tested herein as EGFP fusion forms (reviewed in [1])

**CPP**	**Sequence**	**Reference**
**Buforin 2**	TRSSRAGLQFTPVGRVHRLLRK	[17]
**Transportan**	GWTLNSAGYLLGKINLKALAALAKKIL	[5]
**Transportan 10**	AGYLLGKINLKALAALAKKIL	[18]
**MPG1**	GALFLGWLGAAGSTMGAPKKKRKV	[12]
**MPG2**	GALFLGFLGAAGSTMGAPKKKRKV	[12]
**KALA**	WEAKLAKALAKALAKHLAKALAKALKACEA	[19]
**Pep-1**	KETWWETWWTEWSQPKKKRKV	[6]
**SynB1**	RGGRLSYSRRRFSTSTGR	[20]

In our work, two different MPG sequences were adapted from published works [12] and are shown in Table 1 as MPG1 and MPG2. These MPG sequences do not contain the linker region and thus are modified from the original sequence. The seventh amino acid is tryptophan in MPG1 and phenylalanine in MPG2. This modification of W to F at the seventh position was reported to enhance nuclear localization of the mixed cargo [22]. Both forms, without significant difference, showed effective transduction and exhibited similar cellular distribution and signal intensity (data not shown). Therefore, we used MPG1-EGFP in all the subsequent experiments (indicated as MPG-EGFP thereafter).

### Rapid internalization of MPG-EGFP depends on endocytosis

**Figure 2:**
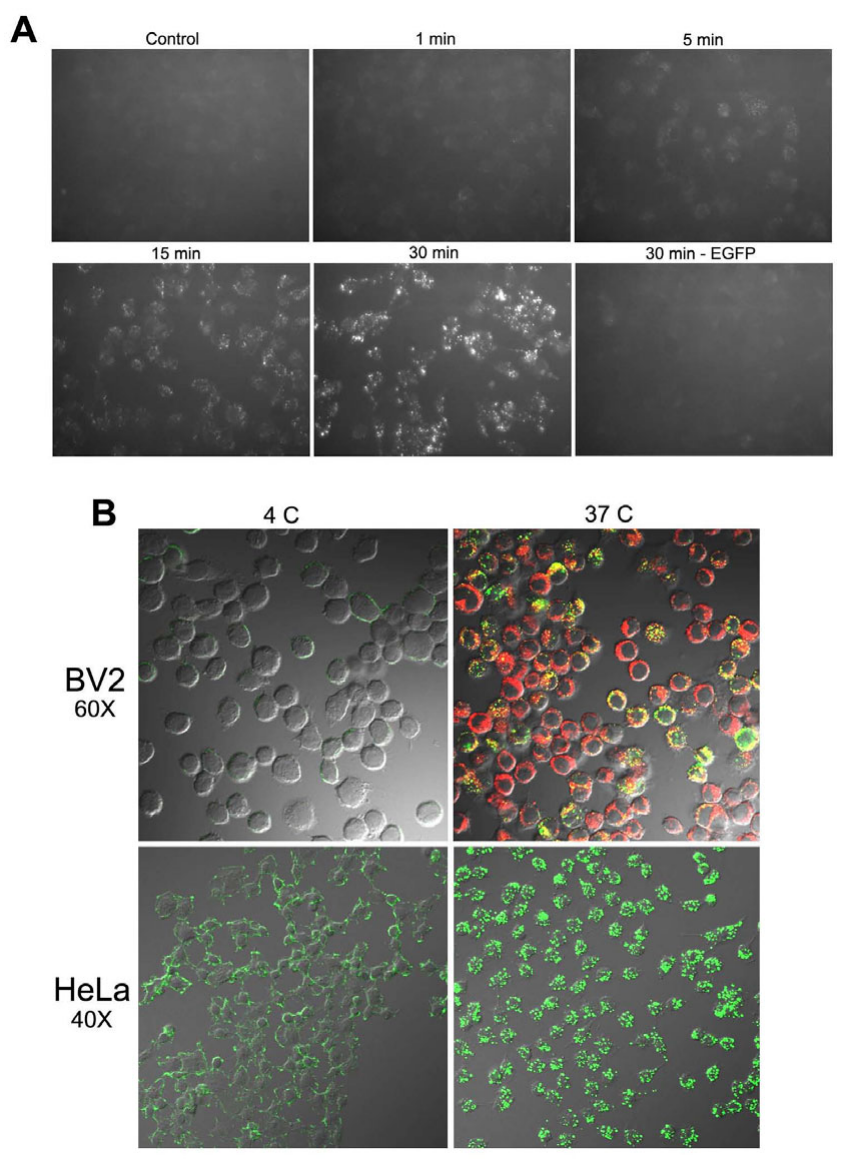
**Internalization of MPG-EGFP occurs rapidly and is inhibited at 4°C.** A. EGFP or MPG-EGFP was added to HeLa cells at 40 μg/ml and cells were fixed with 4% PFA at indicated times after the acid wash. Fixed cells were visualized under an inverted fluorescence microscope. B. 40 μg/ml MPG-EGFP was added to BV2 or HeLa cells in 2-well slide chambers for 30 min, with (BV2) or without (HeLa) 5 μg/ml FM4-64. To maintain 4°C, cells and all reagents were precooled for 20 min on ice. Cells were fixed with 4% PFA for 20 min, mounted, and visualized under a confocal microscope.

Internalization of MPG-EGFP occurs within 1 min and the signal gradually intensifies until about 30 min of incubation (Figure 2A). To eliminate the possibility that MPG-EGFP adheres to the cell surface, we washed MPG-EGFP-treated cells with the acid buffer for 30 sec before observing under a fluorescence microscope in all experiments. The acid wash did not significantly reduce the fluorescence signals of MPG-EGFP, suggesting that the observed signal is not an artifact produced by MPG-EGFP adhering to the cell surface.

To address if the entry of MPG fusion protein depends on endocytosis, we examined the entry of MPG-EGFP at 4°C when general endocytosis is blocked. MPG-EGFP was added to BV2 cells or HeLa cells at 40 μg/ml. BV2 cells were co-treated with FM4-64. As shown in Figure 2B, the uptake of MPG-EGFP was significantly reduced in both cell lines at 4°C, showing only a weak surface binding in some cells. FM4-64 staining was also absent at 4°C in BV2 cells, suggesting efficient blockade of endocytosis in our experimental condition. This result suggests that an energy-dependent endocytic pathway is responsible for the transduction of MPG-EGFP. In the next series of experiments, we investigated which pathway of endocytosis is associated with the uptake of the MPG fusion protein.

### Effects of endocytosis inhibitors on the entry of MPG-EGFP

**Figure 3:**
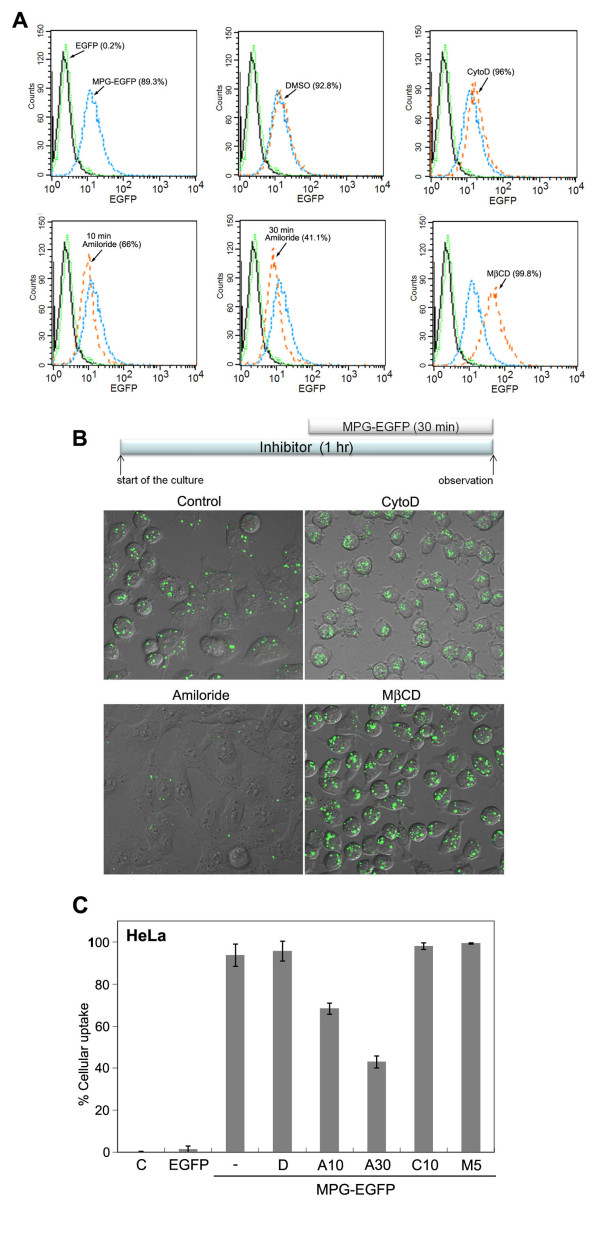
**Transduction of MPG-EGFP is dependent on an amiloride-sensitive endocytic pathway**. A. Flow cytometric analysis of the MPG-EGFP uptake in the presence of various endocytosis inhibitors. The following concentrations were used: 10 μM cytochalasin D, 4 mM amiloride, and 5 mM MβCD. An inhibitor or DMSO (vehicle, 0.2%) was added to HeLa cells 30 min prior to the addition of MPG-EGFP, except for amiloride, which was added 10 min or 30 min prior to the addition of MPG-EGFP. MPG-EGFP at 40 μg/ml was treated to cells for 30 min in the presence of an inhibitor (see the diagram in B.). All cells were washed with the acid buffer before the analysis. Note the decreased MPG-EGFP signal in the amiloride-treated cells. MβCD treatment increased the intensity of EGFP signal significantly. These experiments were repeated at least three times with similar results and one representative set of data is shown. B. Confocal live images of HeLa cells treated with an endocytosis inhibitor as indicated. Photomicrographs were taken at 40X and zoomed in 2X. C. A barogram showing the averaged transduction efficiencies in the presence of various inhibitors. Errors bars represent the standard deviations. C, no protein added; EGFP, 40 μg/ml EGFP protein; MPG-EGFP, 40 μg/ml MPG-EGFP; -, no drug added; D, 0.2% DMSO (vehicle); A10, 4 mM amiloride for 10 min; A30, 4 mM amiloride for 30 min; C10, 10 μM cytochalasin D; M5, 5 mM MβCD.

Endocytosis occurs in cells using multiple pathways, such as clathrin-mediated, caveolae-mediated, lipid rafts-mediated endocytosis, and macropinocytosis [23]. By using various inhibitors of endocytosis, we determined which endocytic pathway is associated with MPG-EGFP uptake. Cytochalasin D, an inhibitor of actin polymerization, blocks actin-dependent macropinocytosis. Amiloride is a sodium channel blocker and is known to block macropinocytosis [24]. Inhibitors were added to the cells 30 min prior to the addition of MPG-EGFP and were maintained at the same concentrations for the duration of the experiments (Figure 3B). When cytochalasin D was added prior to MPG-EGFP treatment, most of cells became round due to the depolymerization of actin filaments but the distinct vesicular pattern of MPG-EGFP remained unchanged. In contrast, 4 mM amiloride treatment effectively reduced MPG-EGFP uptake as shown in Figure 3A. Methyl-β-cyclodextrin (MβCD) depletes cholesterol from the plasma membrane, and is used as an inhibitor of the caveolae-mediated endocytosis and macropinocytosis [25]. Both of these pathways are known to be dependent on cholesterol-rich lipid rafts. Surprisingly, MβCD treatment significantly increased the fluorescence intensity of MPG-EGFP (Figure 3B). Collectively, these results show that the uptake of MPG-EGFP seems to utilize amiloride-sensitive endocytic pathway, but is not dependent on the organization of actin filaments or cholesterol. These experiments were repeated several times with similar results (Figure 3C).

### MPG-EGFP uptake is independent of caveolins but partly dependent on the GTPase activity of dynamins

**Figure 4:**
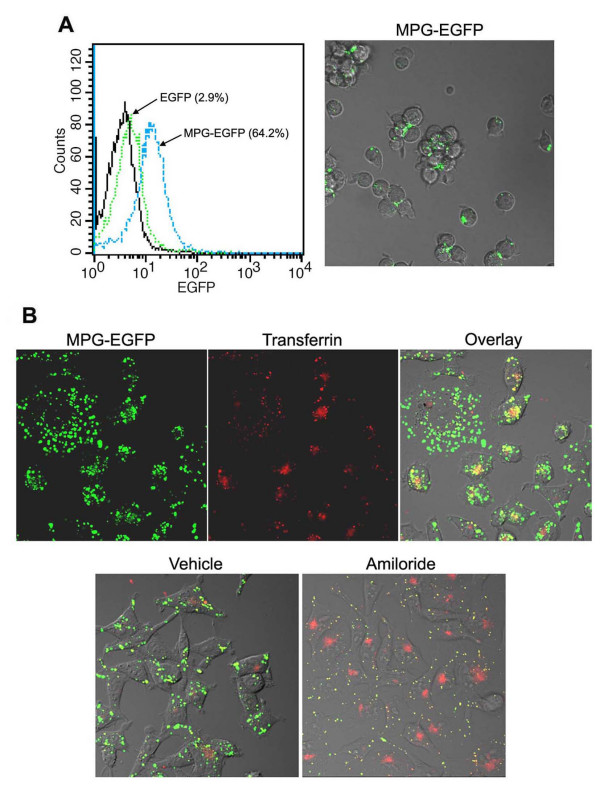
**Transduction of MPG-EGFP is independent on caveolae but may share a pathway with transferrin**. A. Jurkat cells were treated with 40 μg/ml MPG-EGFP for 1 hr. Cells were processed for flow cytometry after the acid wash. In Jurkat cells which lack caveolae, the transduction of MPG-EGFP still occurs. Confocal live image of MPG-EGFP-treated Jurkat cells is shown at 80X. B. Live confocal images showing partial co-localization of transferrin (red) and MPG-EGFP (green) in HeLa cells, generating a yellow fluorescence. Transferrin was added at 50 μg/ml for 30 min along with 40 μg/ml MPG-EGFP. Pre-treatment of amiloride (4 mM for 30 min) significantly reduced MPG-EGFP transduction while transferrin uptake proceeds.

To examine if caveolin-mediated endocytosis is involved in the transduction of MPG-EGFP, we used the human T lymphocyte Jurkat cells which is known to lack caveolin-1 [26]. EGFP or MPG-EGFP was added to the cells at 40 μg/ml for 1 hr and cells were processed for FACS analysis after the acid wash. As shown in Figure 4A, the transduction efficiency of MPG-EGFP in Jurkat cells was above 60%. Although the overall efficiency of MPG-EGFP transduction in Jurkat cells was not as high as in adherent cells, the successful entry of MPG-EGFP shows that this transduction was not dependent on the caveolin-mediated endocytosis.

Dynamin GTPase is required for several types of endocytosis including clathrin-mediated endocytosis [23]. Transferrin is a marker of dynamin-dependent endocytosis. Thus, we first examined if transferrin is co-localized with MPG-EGFP within cells. As shown in Figure 4B, the vesicles containing MPG-EGFP partially overlapped with those of transferrin-Alex Fluor 546, showing a yellow fluorescence. Amiloride treatment did not seem to affect the endocytosis of transferrin but most of MPG-containing vesicles disappeared by this treatment. Some vesicles with both transferrin and MPG were noted.

**Figure 5:**
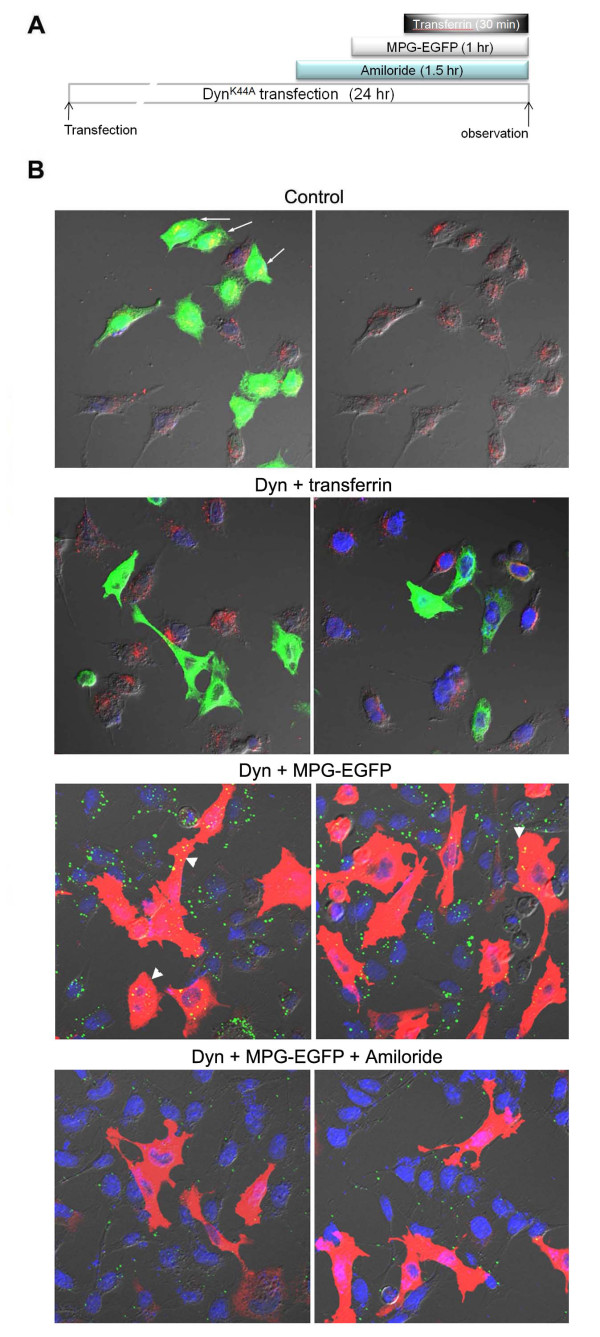
**MPG-EGFP uptake partially depends on the GTPase activity of dynamins**. A. A diagram depicting the experimental scheme of B. B. A mutant form of dynamin I which lacks the GTPase activity (Dyn^K44A^, 1 μg) was transfected into HeLa cells and MPG-EGFP was added at 40 μg/ml for 1 hr. Transferrin was added to cells as a marker of dynamin-dependent endocytic pathway. After washing, cells were fixed and subjected to the immunofluorescence staining with anti-dynamin I antibody. Control, control transfection of 1 μg pEGFP-N1 plasmid showing a high efficiency of transfection. Arrows indicate that transfected cells show normal transferrin localization. Dyn + transferrin, transferrin cannot enter Dyn^K44A^-expressing cells (shown in green), showing efficient blockade of clathrin-mediated endocytosis by overexpression of this mutant form. Dyn + MPG-EGFP, MPG-EGFP was added at 40 μg/ml for 1 hr to Dyn^K44A^-transfected cells. Note the reduced MPG-EGFP localization (arrowheads) in Dyn^K44A^-expressing cells (shown in red). Dyn + MPG-EGFP + amiloride, 30 min pre-treatment of amiloride effectively blocked overall MPG-EGFP uptake. Photomicrographs are shown at 80X.

The observation suggests that dynamins may be partly involved in the transduction of MPG-EGFP. Thus, we tested this hypothesis by using the dominant-negative form of dynamin-1, Dyn^K44A^. Dyn^K44A^, a dominant-negative form blocking the GTPase activity of dynamin I, has been widely used to elucidate the involvement of these proteins in endocytosis [10,27,28]. This form is capable of blocking the GTPase activity of both dynamin-1 and dynamin-2 [29]. Dynamin-1 is a neuron-specific form, while dynamin-2 is ubiquitously expressed [30,31]. We overexpressed Dyn^K44A ^in HeLa cells and added 40 μg/ml MPG-EGFP and 50 μg/ml transferrin (Figure 5A). Expression of Dyn^K44A ^was detected by an antibody specific to dynamin-1 [28]. As shown in Figure 5A, overexpression of Dyn^K44A ^in HeLa cells blocks the entry of transferrin effectively. While MPG-EGFP entry is shown in Dyn^K44A^-expressing HeLa cells, the number of vesicular structures in these cells was notably reduced (arrowheads). Furthermore, amiloride treatment in Dyn^K44A^-expressing HeLa cells almost completely abolished MGP-EGFP uptake, suggesting that the transduction of the MPG fusion protein utilizes unique pathway(s) which is amiloride-sensitive and dynamin-dependent.

### Blockade of a lysosomal pathway intensifies the accumulation of MPG-EGFP in endosomes

**Figure 6:**
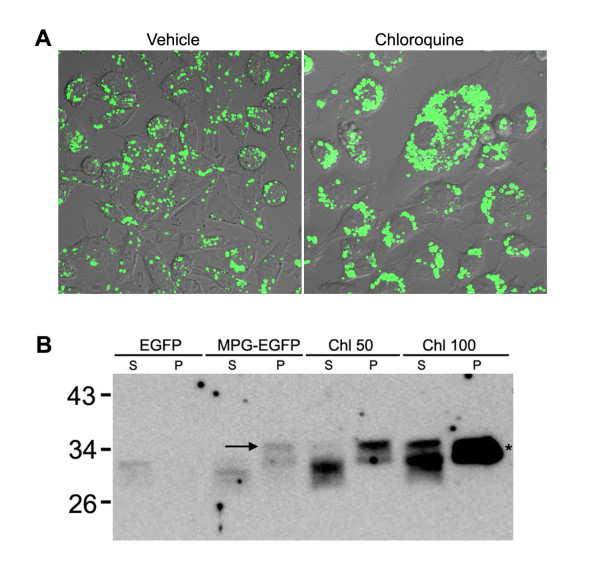
**Chloroquine effectively blocks the endolysosomal pathway of transduced MPG-EGFP**. A. 100 μM chloroquine was added along with 40 μg/ml MPG-EGFP for 17 hr. Note the increased intensity of MPG-EGFP signal and enlarged vesicle. Confocal live images are shown at 80X. B. Vesicle fractionation was performed using HeLa cells treated with MPG-EGFP and chloroquine for 17 hr. Note that the vesicular uptake of MPG-EGFP intensified in the presence of chloroquine, and that the cytosolic fraction also contains a significant amount of MPG-EGFP. EGFP, 40 μg/ml EGFP; MPG-EGFP, 40 μg/ml MPG-EGFP; Chl 50, MPG-EGFP plus 50 μM chloroquine; Chl 100, MPG-EGFP plus 100 μM chloroquine; S, supernatant containing the cytosolic fraction; P, pellet containing the intracellular vesicles. The asterisk indicates that the band is a merged doublet due to the prolonged exposure, and the arrow points the expected size of MPG-EGFP. Western blotting was performed with anti-GFP antibody.

Chloroquine is an inhibitor which blocks the lysosomal pathway of protein degradation [32]. We co-treated chloroquine and MPG-EGFP to HeLa cells for 17 hr to examine if the blockade of the endolysosomal pathway leads to the accumulation of the transduced MPG fusion protein. As shown in Figure 6A, chloroquine-treated cells show enlarged endosomes containing MPG-EGFP. Number of MPG-EGFP-containing vesicles seems to have increased as well. This observation was further explored by the vesicle fractionation experiment. Prolonged treatment of MPG-EGFP showed a lower band representing degradation products. Chloroquine treatment increased the intensity of MPG-EGFP band in the particulate fraction and, notably, the supernatant fraction in 100 μM chloroquine-treated cells contains a much higher level of MPG-EGFP than that in control cells. Collectively, the result shows that the blockade of the lysosomal pathway would increase the transduction efficiency and also lead to the increased release of MPG fusion proteins into the cytosol from the endosomes.

### Uptake of MPG-EGFP in mouse eggs

**Figure 7:**
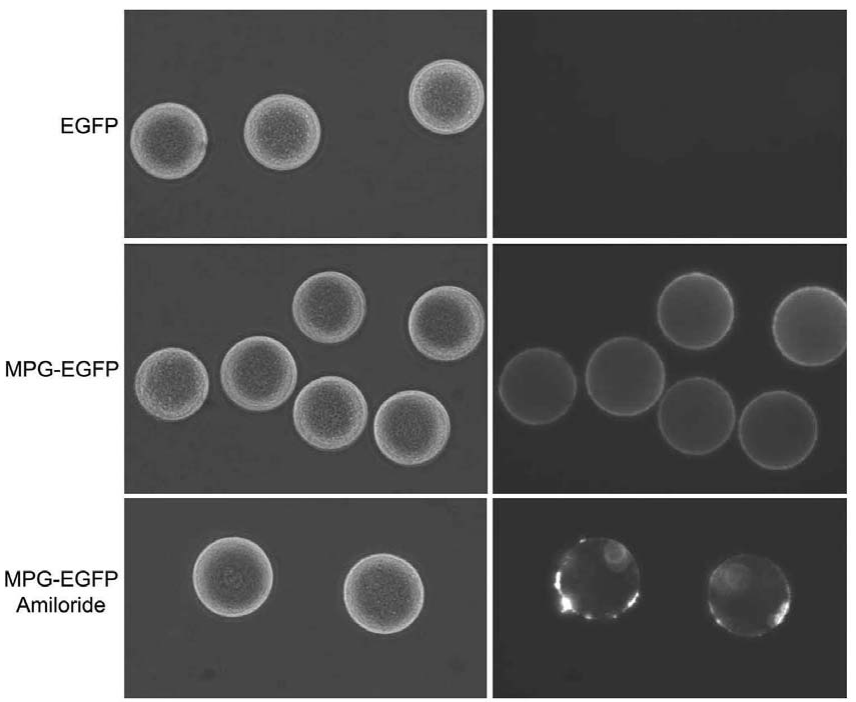
**Successful transduction of MPG-EGFP into the mouse oocytes**. Mature oocytes were obtained from 4-week mouse ovaries, and zona pellucidae were removed by the acid Tyrode solution. EGFP or MPG-EGFP was added to M16 media for 1 hr. Note the strong peripheral EGFP signal in MPG-EGFP treated oocytes. Amiloride, as in HeLa cells, effectively blocked the transduction of MPG-EGFP.

CPP is an excellent tool for a short-term regulation of protein expression. As we showed herein, MPG fusion protein may be a useful tool to deliver proteins to achieve short-term regulation of protein expression. We tested if MPG fusion protein can be used in mammalian eggs for this purpose. So far, the transduction efficiency of any CPP-fusion protein has not been tested in mammalian oocytes. We obtained mouse eggs by the ovary puncture and removed zona pellucidae with the acid Tyrode solution (pH 2). Denuded eggs were treated with control EGFP or MPG-EGFP recombinant protein at 40 μg/ml. As shown in Figure 7, the treatment of EGFP did not give any fluorescence signal, while MPG-EGFP-treated eggs showed strong signal around the ooplasmic periphery. As in the cultured mammalian cells, 4 mM amiloride treatment disturbed the uptake of MPG-EGFP, suggesting that similar endocytic pathway is operative in the mouse egg. Whether MPG fusion protein can be used to deliver proteins to eggs without adverse effects requires further investigation.

## Discussion

In a peptide form, MPG is highly effective in delivering oligonucleotides, plasmid DNA, siRNAs, and peptides via a non-covalent complex formation [12,15,33]. Unlike other CPPs, the uptake of MPG-nucleic acid complex was not inhibited by the treatment of cytochalasin B [34], and this result suggests that the transduction of MPG peptide is independent of macropinocytosis. CPP tag at the N-terminal of a full-length protein would pose an entirely different conformation in the solution. Thus, we aimed to investigate if a similar or distinct cellular pathway of transduction is used for the uptake of the MPG-fusion protein. The macropinocytosis is an actin-dependent endocytic pathway which takes up solutes and fluid by the membrane ruffling and fusion [23]. Several arginine-rich CPPs, such as HIV TAT, Antp, Rev, and VP22 are shown to be dependent on the lipid rafts-mediated macropinocytosis, as their uptake is inhibited by cytochalasin D, MβCD, and amiloride [9,10,35]. TAT-CRE, in a fusion protein form, also used a similar endocytic pathway, and the release of this fusion protein from macropinosomes can be improved by the addition of TAT peptides conjugated with the influenza virus hemagglutinin fusogenic motif [10].

We found that, unlike TAT-fusion protein and arginine-rich CPPs, MPG-EGFP is likely to use a specific endosomal pathway which is sensitive only to amiloride among other endocytosis inhibitors we used. Since the uptake of MPG-EGFP is resistant to actin depolymerization, the macropinocytosis is ruled out. Likewise, caveolae and cholesterol both are not required for the transduction of MPG-EGFP, suggesting that lipid rafts are not associated with the uptake. Notably, the overexpression of the mutant form of dynamin could partially reduce MPG-EGFP uptake in HeLa cells, suggesting that the GTPase activity of dynamins may be involved in the transduction. A recent report showed that the transduction of VP22-EGFP fusion protein was similarly dependent on dynamins, but the uptake of VP22-EGFP is sensitive to MβCD and CytoD [36]. Thus, the transduction pathway is seemingly distinct. Collectively, our results indicate that the transduction of MPG-EGFP seems to utilize a less well-characterized endocytic pathway [23] and suggest that the transduction of MPG-EGFP is dependent on more than singular endocytic pathway, as amiloride and dynamin mutant are both able to reduce the transduction efficiency.

One unique feature of MPG-EGFP transduction is that the sequestration of plasma membrane cholesterol increased cytosolic labeling of this fusion protein (Figure 3). As the cholesterol depletion has a negative effect on the transduction of many CPPs including TAT-fusion protein, this observation further supports that MPG-fusion protein utilizes a distinct endocytic pathway. Enhanced labeling of a fluorescence-tagged octaarginine peptide in the presence of MβCD has been reported and it was suggested that the entry of this peptide depends on more fluidic region of the plasma membrane with less cholesterol [11]. Whether this interpretation can be applied to the case of MPG-fusion protein requires further investigation.

The mechanisms of endocytosis are diverse and new information is continuously being added. The amiloride-sensitive pathway that MPG-EGFP depends on may be somewhat associated with clathrin- and caveolin-independent endocytosis. However, the mechanisms of this pathway mostly remain unknown [37,38]. One novel CPP, namely C105Y, has been reported to use a clathrin- and caveolin-independent endocytosis [39]. The amiloride-sensitive uptake of MPG-EGFP seems to be reminiscent of what was previously described as one type of the endocytic uptake of Chlamydiae [40]. This intracellular bacterium utilizes multiple endocytic pathways, one pathway being the cytochalasin D-resistant and amiloride-sensitive pathway. This is very similar to what we observed herein with the MPG-fusion protein. No other information regarding this pathway is available to delineate the molecular characteristics. A recent report evaluating the entry of the human papillomavirus type 16 showed that this virus utilizes tetraspanin-enriched microdomains, another example of clathrin- and caveolin-independent endocytosis [41]. More elaborate follow-up to dissect the endocytic pathway of MPG-EGFP could serve as a tool to characterize this pathway.

The GTPase dynamin is involved in multiple forms of endocytosis, including caveolae-mediated, clathrin-mediated, and some clathrin- and caveolae-independent endocytic pathway [23]. Therefore, our result showing a partial reduction of MPG-EGFP transduction after the overexpression of the Dyn^K44A ^mutant cannot pinpoint which pathway is utilized by the transduction of this fusion protein. Since the intracellular localization of MPG-EGFP to some extent overlaps with that of transferrin, clathrin-mediated endocytosis may be involved. However, clear dissection on the role for dynamins in CPP transduction warrants further investigation. As in the case of MPG peptide [16], whether the initial interaction of MPG-fusion protein with the cell surface also utilizes negatively charged glycosaminoglycans needs further work. Likewise, studies on the interaction of MPG-fusion protein with lipid membrane will address if MPG-fusion protein bears a similar affinity towards lipids as MPG peptide [22].

We previously showed that MPG-EGFP is effectively transduced into the human amnion-derived mesenchymal stem cells [42]. This system may also be applicable to the oocyte system as a short-term manipulation of protein expression, as we show herein.

## Conclusion

In this work, we show that MPG fusion protein utilizes an endocytic pathway(s) which is amiloride-sensitive and partially dynamin-dependent, which is entirely different from other widely used CPPs. Thus, we suggest that the MPG fusion protein could be further developed as a novel tool of "protein therapeutics", with potentials to be used in various cell systems including mammalian oocytes. Furthermore, the MPG fusion protein system can be used to target and deliver cargos to certain subcellular compartments. And it also may be a useful tool to explore the mechanism of previously uncharacterized type of endocytosis.

## Methods

### Cell culture

AN_3_CA human uterine adenocarcinoma cells, HeLa, 293T, NIH3T3, and BV2 cells were purchased from American Type Culture Collection (Rockville, MD, USA) and were cultured in DMEM supplemented with 10% FBS (Invitrogen, Carlsbad, CA, USA). Jurkat cells were cultured in RPMI 1640 supplemented with 10% FBS.

### Expression and purification of CPP-EGFP fusion proteins

Full-length EGFP was amplified using pEGFP-N1 vector (Invitrogen) as a template and cloned into XhoI and BamH1 sites of the pET15b vector (Novagen, Madison, WI, USA). Transportan, MPG1, MPG2, and KALA fragments were amplified by Klenow reaction, and then cloned into NdeI and XhoI site in pET15b-EGFP. Other CPP sequences were included in the 5' primers, directly amplified using pET15b-EGFP as a template, and cloned into the pGEM-T-Easy vector (Promega, Madison, WI, USA). After sequence verification, fusion constructs were released and cloned into NdeI and BamHI sites of the pET15b-EGFP. Primer information is available upon request.

Purification of CPP-fused recombinant proteins were performed as described previously [43]. An overnight culture of 1 ml (BL21(DE3), Novagen) was added to 100 ml LB medium containing 100 μg/ml of ampicillin and incubated at 37°C and 200 rpm to an OD_600 _of 0.5. Protein expression was induced by adding 1 ml of 100 mM IPTG solution and the culture was incubated for 6 h at 25°C and 200 rpm. The cells were harvested by centrifugation (10 min, 4000 rpm, 4°C) and the supernatant was discarded. The cell pellet was resuspended in 4 ml of the lysis buffer (50 mM NaH_2_PO_4_, 300 mM NaCl, 10 mM imidazole, pH 8.0), and lysozyme was added to 1 mg/ml. Incubation on ice for 45 min was followed by a freeze-thaw cycle at -20°C and room temperature. The viscous lysate was passed several times through a sterile 20-gauge syringe needle and centrifuged (10 min, 10,000 rpm, 4°C). The supernatant was separated from the cell debris. MPG1, MPG2, Buforin 2 and SynB1 fusion proteins and EGFP were yielded as soluble fractions and purified in a native state. Ni-NTA agarose resin (1 ml, 50% w/v slurry, Qiagen Inc., Valencia, CA) was added to the supernatant and the mixture was shaken at 25°C for 1 h. The lysate-Ni-NTA mixture was loaded on a Poly-Prep column (Bio-Rad, Hercules, CA, USA), drained, and then washed once with 2 ml of the lysis buffer and three times with 4 ml of the wash buffer (50 mM NaH_2_PO_4_, 300 mM NaCl, 20 mM imidazole, pH 8.0). The proteins were eluted from the column with the elution buffer (50 mM NaH_2_PO_4_, 300 mM NaCl, 250 mM imidazole, pH 8.0). The buffer of the protein solution was exchanged to 1 ml of PBS buffer using a centrifugal device (Amicon Ultra-15, Millipore, Billerica, MA, USA). Transportan, Transportan 10, KALA, and Pep-1 fusion proteins were yielded as insoluble inclusion bodies and purified under a denaturating condition. Ni-NTA agarose resin (1 ml, 50% w/v slurry) was added to the cell debris in 2 ml of 8 M urea solution and the mixture was stirred at 25°C for 1 hr. The lysate-Ni-NTA mixture was loaded on a Poly-Prep column (Bio-Rad), drained, and then washed three times with 4 ml of the buffer C (100 mM NaH_2_PO_4_, 10 mM Tris-Cl, 8 M urea, pH 6.3) and two times with 1 ml of the buffer D (100 mM NaH_2_PO_4_, 10 mM Tris-Cl, 8 M urea, pH 5.9). The proteins were eluted from the column with the buffer E (100 mM NaH_2_PO_4_, 10 mM Tris-Cl, 8 M urea, pH 4.5). The protein solution was 50-fold diluted with the renaturation buffer (35 mM KCl, 2 mM MgCl_2_, 50 mM Tris-HCl, 1 mM DTT, pH 7.5) and incubated at 4°C overnight. The diluted solution was concentrated and the buffer was exchanged to 1 ml of PBS buffer using a centrifugal device.

### Fluorescence microscopy

Cells were seeded onto a 12-well plate at the density of 1 × 10^5 ^a day before the experiment. Cells treated with CPP-EGFP or control EGFP at indicated concentrations were washed with HBSS (Invitrogen), with the acid wash buffer (0.2 M glycine, 0.15 M NaCl, pH 3.0) for 30 sec [44], and then with HBSS again. The cellular uptake of CPP-EGFP was visualized in HBSS by Zeiss AxioVert200 inverted microscope equipped with GFP and DAPI filters (Carl Zeiss, Jena, Germany). In some experiments, live images were obtained by using Olympus FV1000 spectral confocal microscope (Olympus, Tokyo, Japan) equipped with a warm plate. In these experiments, cells were seeded onto a glass-bottom plate (SPL Lifesciences, Pocheon, Korea) and stained with 5 μg/ml FM4-64 (Invitrogen) for 1 min. To observe MPG-EGFP uptake at different time points, MPG-EGFP-treated cells were fixed with 4% paraformaldehyde (PFA) in PBS at 1 min, 5 min, 15 min, and 30 min after transduction, washed with PBS, and visualized in HBSS under an inverted microscope. Transferrin-Alexa Fluor 546 conjugate and LysoTracker Red DND-99^® ^were purchased from Invitrogen.

### Vesicle fractionation

A vesicle-enriched fraction and a cytosolic fraction from MPG-EGFP-treated cells were prepared as follows. HeLa cells on a 100 mm culture dish were treated with 40 μg/ml MPG-EGFP for 1 hr and washed with HBSS and the acid wash buffer. The lysis buffer (10 mM Tris-Cl [pH 7.4], 320 mM sucrose, 150 mM NaCl, 1 mM EDTA) of 400 μl were added. Cells were then homogenized with a Dounce homogenizer gently and centrifuged at 1000 × g for 10 min at 4°C. The supernatant was then centrifuged at 100,000 × g for 1 hr at 4°C. The resultant supernatant is the cytosolic fraction, and the pellet is the particulate fraction containing vesicular structures within cells. These samples were run on 10% SDS-PAGE gel, blotted onto a nitrocellulose paper, and subjected to Western blotting using anti-GFP antibody (Young-In Frontier, Seoul, Korea).

### Inhibition of endocytosis

To block general endocytosis at 4°C, cells were plated onto a 2-well slide chamber and precooled on ice for 20 min. All reagents were also precooled on ice. MPG-EGFP at 40 μg/ml was added to the cells and incubated for 30 min on ice. After 30 min, cells were briefly fixed with 4% PFA in PBS and slides were mounted using Profade Gold antifade reagent (Invitrogen) to be visualized under an inverted microscope. Chloroquine, cytochalasin D, amiloride, and methyl-β-cyclodextrin were purchased from Sigma-Aldrich (St. Louis, MO, USA) and used at indicated concentrations.

### Flow Cytometric Analysis

MPG-EGFP-treated cells were washed with HBSS and the acid wash buffer, and then trypsinized with 0.01% trypsin-EDTA solution for 10 min. Cells were resuspended in DMEM supplemented with 10% FBS, and washed with PBS containing 3% FBS. After washing, cells were analyzed on FACSCalibur flow cytometer (BD Biosciences, Franklin Lakes, NJ, USA) equipped with Cell Quest Pro software (BD Biosciences).

### Transfection of Dynamin^K44A^

For transfection of dynamin-1 K44A mutant cDNA [27], Fugene HD (Roche, Indianapolis, IN, USA) and 1 μg of plasmid DNA were mixed in the serum-free media at 4:1 ratio and incubated for 15 min at RT. The mixture was added to HeLa cells seeded in 2-well slide chambers. Twenty-four hours later, 40 μg/ml MPG-EGFP was treated to cells for 1 hr and processed for the immunofluorescence staining [42]. Transferrin at 50 μg/ml was added to these cells for 30 min prior to the fixation. Cells were fixed with 4% PFA, nonspecific antiserum binding was blocked with 2% BSA/PBS for 60 min. Cells were incubated for 1 hr with anti-dynamin I mouse monoclonal antibody diluted to 1:100 in 2% BSA/PBS. Following three washes with 2% BSA/PBS, a goat anti-mouse secondary antibody conjugated with Alexa 568 or Alex 488 (Invitrogen, 1:250 in 2% BSA/PBS) was applied for 30 min. Subsequently slides were washed with 2% BSA/PBS and nuclei were stained with TO-PRO-3-iodide (Invitrogen, 1:500) for 20 min. After a final wash with PBS, coverslips were mounted using ProLong Gold antifade reagent (Invitrogen). Specimens were examined by using Olympus Fluoview™ FV1000 confocal microscope (Olympus, Japan) and analyzed using the software Fluoview version 1.5, a platform associated with the confocal microscope. The primary antibody used is specific for the neuron-specific dynamin-1 (BD Biosciences, San Jose, CA, USA) as the epitope at the C-terminal region is variable in Dynamin-2 [30].

### Confocal laser scanning microscopy

Cells were split and seeded on the poly-*L*-lysine coated cover slips placed in a 6-well plate a day before the experiment. Cells were treated with CPP-EGFP or EGFP for one hour and intracellular localization of EGFP was observed as follows. Cells were washed twice with PBS and fixed for 10 min in 4% PFA in PBS. Cells were washed with PBS three times, counterstained with TO-PRO-3-iodide in PBS (1:500, Invitrogen) for 20 min, and rinsed three times in PBS. Coverslips were mounted onto sections with ProLong Gold mounting media (Invitrogen) and sealed with nail polish. Images were obtained using the Olympus Fluoview™ FV1000 confocal microscope. For live imaging, unfixed cells on a glass-bottom plate were placed onto a warm plate and images were obtained for 10–20 min at 20 sec interval.

### Oocyte handling

Five-week old virgin CD-1 female mice were purchased from Orient-Bio (Gyunggi-do, Korea). Mice were maintained in accordance with the policies of the Institutional Animal Care and Use Committee in Konkuk University. Female mice were injected i.p. with 5 I.U. pregnant mare's serum gonadotropin (PMSG) (Sigma) to stimulate the growth of preovulatory follicles. Forty-eight hrs later, ovaries were taken from these mice and were punctured with fine forceps to release fully grown oocytes. Cumulus cells were removed by repeated pipetting and zona pellucidae were digested with the acid Tyrode solution [45]. Purified EGFP or MPG-EGFP in M16 media was added to the denuded oocytes at the indicated concentration and EGFP signal was visualized under an inverted fluorescence microscope.

## Authors' contributions

SJK, KH, SJ, and JEL performed experiments and interpreted data. SJK organized experiments and prepared figures. SP and YPC participated in research design and manuscript writing. HJL supervised the overall experiments and wrote the manuscript. All authors read and approved the final manuscript.

## Supplementary Material

Additional file 1 **Live images of HeLa cells treated with 40 μg/ml MPG-EGFP and 5 μg/ml FM4-64**. Cells on a glass-bottom plate were placed onto a warm plate and images were obtained for 50 min at 20 sec interval. Images were obtained using the Olympus Fluoview™ FV1000 confocal microscope. Click here for file
